# Collision of a Pancreatic Neuroendocrine Tumor and Intra-ampullary Carcinoma: A Case Report and Review of the Literature

**DOI:** 10.7759/cureus.73829

**Published:** 2024-11-16

**Authors:** Andres R Latorre-Rodriguez, Orlando Ricaurte Guerrero, Carlos A Cano Muñoz

**Affiliations:** 1 School of Medicine, Universidad Nacional de Colombia, Bogota, COL; 2 Norton Thoracic Institute, St. Joseph's Hospital and Medical Center, Phoenix, USA; 3 Department of Pathology, Universidad Nacional de Colombia, Bogota, COL; 4 Department of Pathology, Hospital Universitario Nacional de Colombia, Bogota, COL; 5 Department of Pathology, Hospital Universitario Hernando Moncaleano Perdomo de Neiva, Neiva, COL

**Keywords:** adenocarcinoma, biliary system neoplasms, gastrointestinal oncology, hepato biliary cancers, hepato-biliary-pancreatic surgery, malignant pancreatic cancer, neuroendocrine tumors, pancreatic neoplasms, synchronous neoplasms

## Abstract

This manuscript reports the case of a 75-year-old patient presenting with a collision tumor consisting of a pancreatic neuroendocrine tumor and intra-ampullary adenocarcinoma, which manifested with obstructive jaundice and was treated with primary surgical cytoreduction. Additionally, a bibliographic search of original articles was performed in the Medical Literature Analysis and Retrieval System Online (MEDLINE; via PubMed) and the Latin American and Caribbean Literature on Health Sciences (LILACS) databases to review the literature on pancreaticobiliary collision tumors. Currently, information regarding pancreatic and bile duct collision tumors is limited due to their very low incidence. There are only occasional case reports in the literature. The present case demonstrates that the definitive diagnosis is based on histopathological findings alone; furthermore, it is challenging to distinguish between a mixed neuroendocrine-nonneuroendocrine neoplasm (MiNENs) and a collision tumor with a neuroendocrine component.

## Introduction

Collision tumors are defined as the coexistence of at least two different malignant neoplasms in the same anatomic location without a transition zone or a clear mixture between them [1, 2]. In the pancreas, biliary tract, and ampulla of Vater, collision tumors are rare, corresponding to 0.06%-0.2% of all neoplasm. The usual clinical presentation of collision tumors, regardless of their histology, is an obstructive jaundice [3]. The prognosis of these tumors is usually poor, resulting in short overall survival, likely due to their rarity and late diagnosis. The primary treatment is often surgical cytoreduction and adjuvant chemotherapy [4].

## Case presentation

This was the case of a 75-year-old man who had sought consultation several times for obstructive jaundice and recurrent cholangitis over one year. The patient received symptomatic and antibiotic management, and during one of the episodes a significant stenosis of the common bile duct was documented, necessitating endoscopic management with retrograde cholangiopancreatography and external derivation.

**Figure 1:**
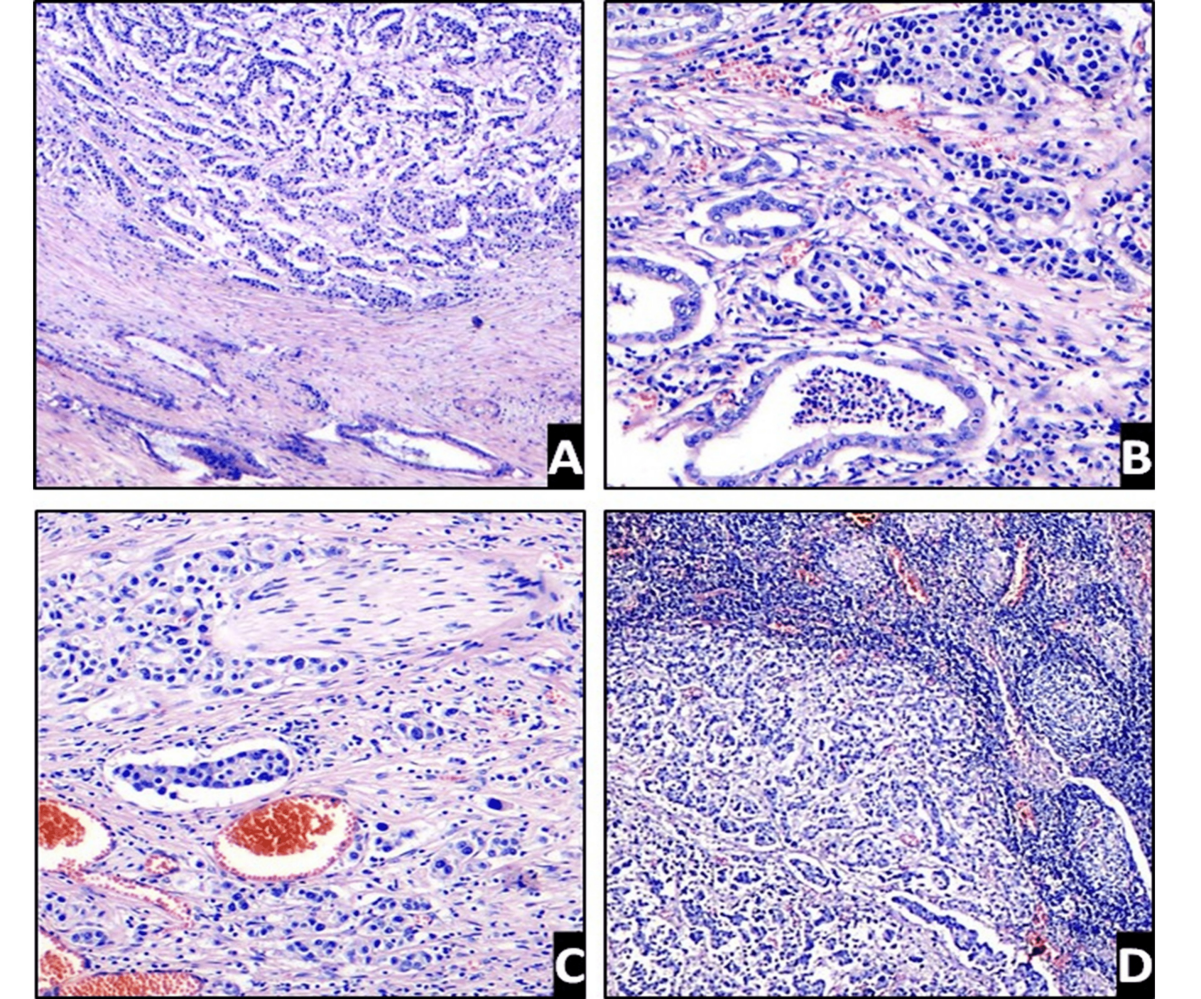
Pathological findings (H&E staining) A. The upper area shows diffuse neoplastic proliferation of small cells arranged in nests and trabeculae reminiscent of an organoid pattern, and the area at the bottom shows a malignant tumor with biliary epithelial differentiation formed by ductal structures (H&E 4x); B. Detail of the closest areas between the two histological tumor types (H&E 10x); C. Evidence of perineural and lymphovascular invasion of the neuroendocrine tumor (H&E 40x); D. Nodal involvement by the neuroendocrine tumor with accompanying reactive follicular hyperplasia (H&E 10x).

 During the last episode, a lesion in the head of the pancreas was identified via echo-endoscopy and required urgent trans-duodenal sphincteroplasty, biliary tract exploration, T-tube placement, and laparoscopic cholecystectomy. Once the acute phase was resolved, nuclear magnetic resonance imaging (MRI) was performed, confirming the existence of a mass in the head of the pancreas and multiple abnormal peripancreatic lymph nodes. Subsequently, the patient was scheduled for primary cytoreduction (i.e., pancreatoduodenectomy). The intraoperative findings included a significant dilatation of the common bile duct measuring 2 cm in diameter, the presence of two plastic stents into the lumen (i.e., from previous endoscopic intervention), and prominent periportal, duodenal, and transverse mesocolon retractions in the head of the pancreas, and an irregular, indurated mass of approximately 3 cm in diameter was observed with significant edema but without evidence of infiltration into the superior mesenteric vein. The body and the tail of the pancreas showed moderate atrophy, with dilatation of 7 mm of the Wirsung's duct at the level of the isthmus and multiple peripancreatic pathological lymph nodes in the anterior and posterior aspect (i.e., 1-3 cm in diameter). No ascites, carcinomatosis, or hepatic metastatic lesions were identified. The surgical specimen consisted of a segment of gastric antrum, duodenum, and the head of the pancreas with a plastered appearance. At the macroscopic assessment, two plastic stents were identified in the ampulla and the intrapancreatic bile duct; moreover, at 1 cm from the resection margin of the common bile duct, there was a yellowish-white mass of 3 x 3 x 2 cm with irregular borders and infiltrating the duodenal wall. The microscopic assessment of the pancreas revealed a tumor with an organoid appearance, consisting of nests, trabeculae, and cords of polygonal cells, homogeneous, with scant eosinophilic cytoplasm and rounded nuclei with slight atypia, presenting up to 15 mitotic figures per 10 fields of 40x, discrete foci of necrosis with invasion of the duodenal wall and the biliary tract as well as perineural infiltration and discrete lymphatic invasion. In addition, an intra-ampullary malignant epithelial tumor with biliary differentiation was identified. This tumor was composed of ductal structures of cylindrical cells with scarce cytoplasm with accentuated nuclear atypia and atypical mitotic figures extending to the adjacent pancreatic tissue with marked desmoplastic reaction; moreover, a focal in-situ component and lymphatic and perineural invasion were documented. Twenty-five lymph nodes were identified, 21 presenting evidence of metastasis of the neuroendocrine tumor (Figure 1).

**Figure 2:**
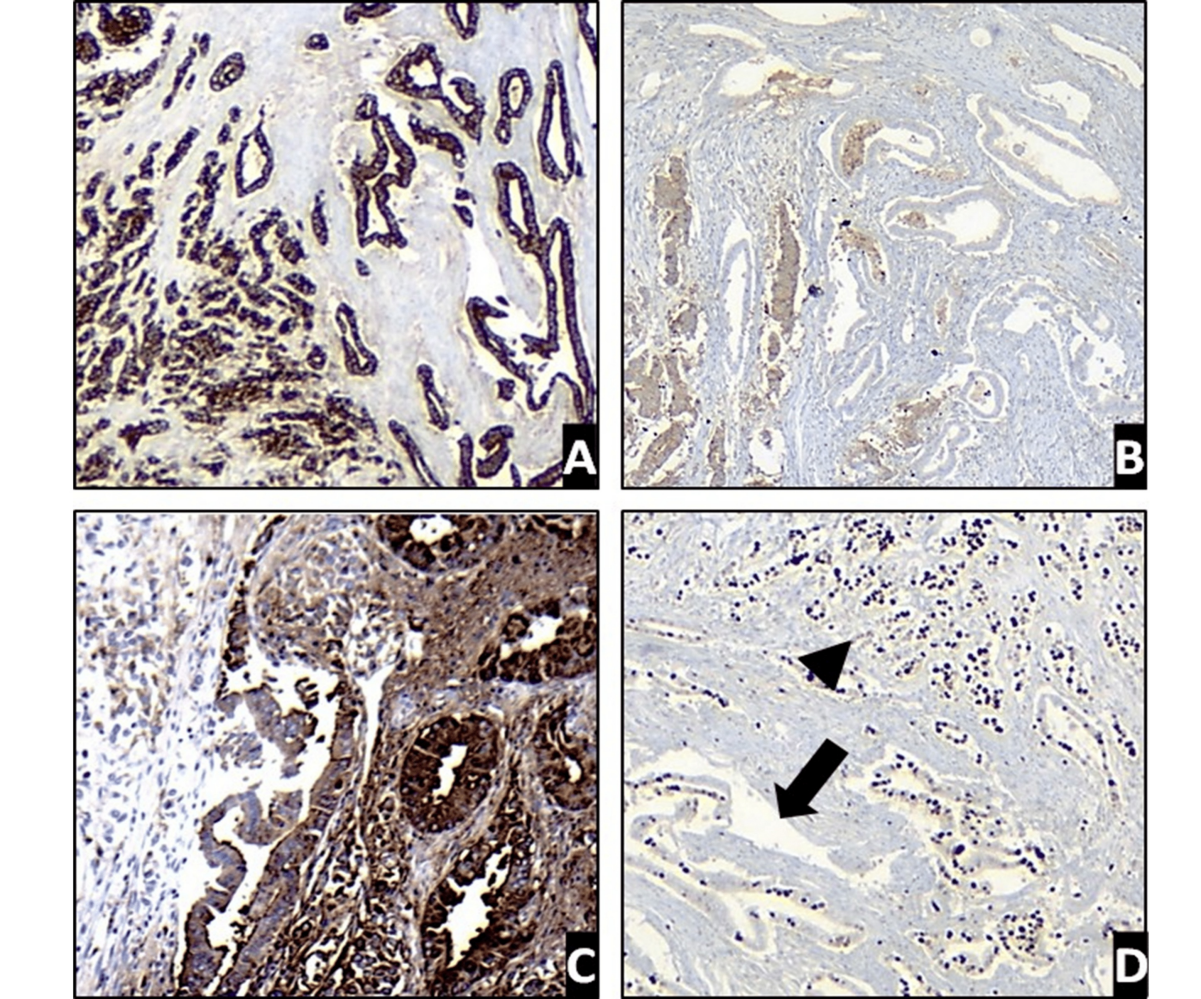
Immunohistochemistry reports A. CK AE1/AE3 expression in neoplastic cells shows a clear differentiation of the neuroendocrine component on the left side and evidence of tubular structures with biliary differentiation on the right side (4x); B. Synaptophysin expression in the neuroendocrine component (4x); C. Strong and diffuse expression of CA19.9 in biliary adenocarcinoma (10x); D. Cell proliferation index of 90% in the neuroendocrine component (arrowhead) and 70% in the biliary component (arrow) (4x).

The immunohistochemistry assessment of the pancreatic neoplasm revealed neuroendocrine features, with diffuse reactivity for synaptophysin, and focal reactivity for chromogranin and CK19, and negative staining for CA 19.9. On the other hand, the tumor with biliary differentiation exhibited intense diffuse reactivity for CK AE1/AE3 and CK19 but lacked immunoreactivity for synaptophysin and chromogranin. The cell proliferation index determined with Ki-67 was 90% in the neuroendocrine-like component and 70% in the adenocarcinoma component with biliary differentiation (Figure 2).

During the surgical procedure, there was significant blood loss (~1 liter) requiring red blood cell transfusion, vasopressor support, and admission to the intensive care unit. The patient's clinical evolution was favorable; however, the patient presented febrile peaks with elevated acute phase reactants, and further radiological studies documented a small subhepatic abscess that was treated with parenteral antibiotic management using piperacillin-tazobactam. The patient was discharged from the hospital on postoperative day 13. In the postoperative period, the patient had an overall adequate recovery but developed diarrhea and appetite loss secondary to exocrine pancreatic insufficiency, for which oral pancreatic enzymes were initiated. The patient was then referred to another institution to complete an adjuvant chemotherapy regimen. The hepatobiliary surgery team at our institution followed up with the patient for a total of five months.

## Discussion

We conducted a non-structured literature search using Medical Subject Headings (MeSH) and free-text terms (e.g., "Pancreatic collision tumor", "Pancreatic adenocarcinoma", "Pancreatic neuroendocrine collision tumor") in the Medical Literature Analysis and Retrieval System Online (MEDLINE; via PubMed) and the Latin American and Caribbean Literature on Health Sciences (LILACS) databases for studies published between January 1971 and December 2020.. Only published original articles, review articles, case and case series reports, and meta-analyses were considered for further review. A total of 37 search results related to collision tumors of the pancreas and biliary tract were obtained. However, 27 publications were excluded as they addressed other histologic tumor types or different types of publications. Only 10 manuscripts-nine case reports and one retrospective case series that included a survival analysis-were published during the specified period. Additionally, other relevant literature was reviewed to enhance the discussion [1-14].

**Table 1:** Summary of isolated cases reported between 1996-2020 CT scan: computed tomography scan; F: female; FNA: fine-needle-aspiration; GIST: gastrointestinal stromal tumor; M: male; US: ultrasound

Author, year	Age (years)	Sex	Clinical presentation	Tumor location	Radiological evaluation	Surgical treatment	Tumoral components (histopathology)
Williams et al., 1996 [3]	58	M	Weight loss and jaundice	Ampulla of Vater	Abdominal US and CT scan	Whipple's procedure	Moderately differentiated adenocarcinoma + carcinoid tumor
Marco et al., 2007 [10]	64	M	Jaundice and constitutional syndrome	Periampullary	Abdominal US and CT scan	Pancreaticoduodenectomy with pyloric preservation	Well-differentiated adenocarcinoma + carcinoid tumor
Sastry et al., 2014 [8]	81	M	Jaundice and constitutional syndrome	Periampullary	Magnetic resonance	Pancreaticoduodenectomy with pyloric preservation	Poorly differentiated adenosquamous carcinoma + moderately differentiated ductal adenocarcinoma + well-differentiated neuroendocrine tumor with cystic degeneration
Izumi et al., 2015 [9]	70	M	Jaundice	Periampullary	Abdominal US, CT scan, and magnetic resonance	Pancreaticoduodenectomy with pyloric preservation	Moderately differentiated tubular adenocarcinoma + well-differentiated tubular adenocarcinoma
Chan et al., 2015 [11]	73	F	Abdominal discomfort	Periampullary	CT scan and PET scan	Whipple's procedure + ileal resection and biopsy of the liver lesions	Moderately differentiated adenocarcinoma + low-grade neuroendocrine tumor
Serafini et al., 2017 [2]	69	F	Weight loss, epigastric pain, and fatigue	Periampullary	Abdominal US and CT scan	Total pancreatectomy and excision of the jejunal lesion	Ductal adenocarcinoma + metastatic neuroendocrine tumor + GIST
Costa et al., 2017 [12]	56	M	Asymptomatic	Pancreas	Endoscopic US and CT scan	None, clinical observation alone	Intraductal papillary mucinous neoplasm of the pancreas + neuroendocrine tumor (diagnosis done using FNA)
Huang et al., 2018 [13]	48	M	Right lower quadrant pain, diarrhea, and vomiting	Pancreas	CT scan	None, chemotherapy alone	Adenocarcinoma + lipid-rich neuroendocrine tumor (diagnosis done using FNA)
Wang et al. 2018 [1]	51	F	Right upper quadrant abdominal pain	Periampullary	Endoscopic US and CT scan	Whipple's procedure	Ductal adenocarcinoma + well-differentiated neuroendocrine tumor

Multiple primary neoplasms (MPN) are defined according to the criteria proposed by Warren and Gates in 1932 [5], which emphasize that i) each component must present evident characteristics of malignancy, ii) correspond to different histological types, and iii) the possibility that one of the neoplasms is the result of metastatic disease of the other must be ruled out. Based on the timing of diagnosis, the second neoplasm is considered synchronous if diagnosed within six months or metachronous if diagnosed after six months [5,6]. Recently, the World Health Organization (WHO) introduced the concept of "collision tumor" in the context of synchronous neoplasms. This concept corresponds to two malignant neoplasms of different histogenesis in the exact anatomical location without a transition zone or mixture between them [1], histological features that were evident in the present case. Neoplasms of the pancreas, biliary tract, and ampulla are a small fraction of cancer incidence worldwide, accounting for 2.3-3.4 cases per 100,000 inhabitants [7]. In general, it is estimated that about 5%-10% of all cancer patients may present MPN [8]. Pancreaticobiliary tumors are exceptional, and there is not enough information in the literature to establish their prevalence [1,2,8]. Nevertheless, it is reported that they could correspond to 0.06%-0.2% of the neoplasms at this anatomical location [2]. So far, it has not been possible to determine a significant difference in the incidence by gender, race, or other condition that may represent a risk factor; however, the mean age of presentation is approximately 60.4 years [4]. The most frequent clinical presentation is obstructive jaundice likely due to its location [3,8-10]. Other presentations include constitutional symptoms [2,8], abdominal pain, and emesis, albeit less commonly [1,11,13]. Although abnormal elevation of CA19-9 as a serum marker has been documented in some cases [4], there is no available specific diagnostic biomarker (radiological or biological) to determine the biological nature of a pancreaticobiliary collision tumor. Therefore, the diagnosis must be confirmed histopathologically. Most ampullary carcinomas are predominantly gland-forming, such as intestinal-type adenocarcinoma and pancreaticobiliary-type adenocarcinoma. In contrast, non-glandular growth pattern carcinomas including mucinous, poorly cohesive, medullary, adenosquamous, high-grade neuroendocrine, and undifferentiated carcinomas are less common [14]. Neuroendocrine tumors of the gastrointestinal and hepatopancreaticobiliary tract are classified as well or poorly differentiated. The histological grading of these tumors is based on mitotic count and Ki-67 proliferation index [14]. Moreover, there is a particular category of mixed neuroendocrine-non-neuroendocrine neoplasms (MiNENs), consisting of both a neuroendocrine component independent of its grading and another histologic component that must constitute at least 30% of the tumor, which can be intermingled. In the small bowel and ampulla, the most frequent combination is tubular adenocarcinoma with neuroendocrine carcinoma.

Furthermore, Niu et al. reported a series of 10 cases of pancreatic and periampullary collision tumors [4], with the most common histopathologic pattern being intraductal papillary mucinous neoplasms of the pancreatic head and tubular adenocarcinomas of the distal common bile duct. Only two cases shared the same histological features as those in this report [4]. Notably, the prognosis for collision tumors in this anatomical location is poor, even following radical resection and adjuvant chemotherapy. Nevertheless, radical resection combined with adjuvant chemotherapy, typically with gemcitabine for resectable tumors, remains the recommended treatment [4]. The carcinogenic factors and mechanisms underlying collision tumors are still unknown, highlighting the need for further research to identify risk factors, understand mechanisms of local recurrence and distant metastasis, and improve overall survival.

## Conclusions

Pancreatic and bile duct collision tumors are extremely rare, with only a limited number of reports and case series in the literature. Furthermore, histopathological diagnosis can be challenging due to the difficulty in distinguishing between MiNENs and collision tumors with a neuroendocrine component. Despite their poor and unpredictable prognosis, prognosis, surgical resection remains the cornerstone of treatment. Given the complex nature of these neoplasms, an interdisciplinary approach is recommended, including collaboration between pathology, radiology, hepatobiliary surgery, and clinical oncology to ensure accurate diagnosis and management.
